# Cu[B_2_(SO_4_)_4_] and Cu[B(SO_4_)_2_(HSO_4_)]—Two Silicate Analogue Borosulfates Differing in their Dimensionality: A Comparative Study of Stability and Acidity

**DOI:** 10.1002/anie.201803395

**Published:** 2018-05-16

**Authors:** Jörn Bruns, Maren Podewitz, Klaus R. Liedl, Oliver Janka, Rainer Pöttgen, Hubert Huppertz

**Affiliations:** ^1^ Leopold-Franzens-Universität Innsbruck Institut für Allgemeine Anorganische und Theoretische Chemie Innrain 80–82 6020 Innsbruck Austria; ^2^ Westfälische Wilhelms-Universität Münster Institut für Anorganische und Analytische Chemie Corrensstrasse 30 48149 Münster Germany

**Keywords:** borosulfates, crystal structure, sulfuric acid

## Abstract

Borosulfates are an ever‐expanding class of compounds and the extent of their properties is still elusive. Herein, the first two copper borosulfates Cu[B_2_(SO_4_)_4_] and Cu[B(SO_4_)_2_(HSO_4_)] are presented, which are structurally related but show different dimensionalities in their substructure: While Cu[B_2_(SO_4_)_4_] reveals an anionic chain, ${}_{\infty }^{1}$ [B(SO_4_)_4/2_]^−^, with both a twisted and a unique chair conformation of the B(SO_4_)_2_B subunits, Cu[B(SO_4_)_2_(HSO_4_)] reveals isolated [B_2_(SO_4_)_4_(HSO_4_)_2_]^4−^ anions showing exclusively a twisted conformation. The complex anion can figuratively be obtained as a cut‐out from the anionic chain by protons. Comparative DFT calculations based on magnetochemical measurements complement the experimental studies. Calculation of the p*K*_a_ values of the two conformers of the [B_2_(SO_4_)_4_(HSO_4_)_2_]^4−^ anion revealed them to be more similar to silicic than to sulfuric acid, highlighting the close relationship to silicates.

During the recent years, borosulfates have gained increasing interest, owing to their close structural relation to silicates.^1^ The structural diversity of borosulfates is supposed to be as complex as those of silicates; however, mainly unexplored, despite their potential application properties. The hitherto obtained anionic subunits range from separated anions to extended 3D networks. Numerous examples have anionic chains of corner‐linked (SO_4_) and (BO_4_) tetrahedra, for example, *A*[B(SO_4_)_2_] (*A*=Ag^+^, Na^+^, K^+^, NH_4_^+^, H_3_O^+^).^2^ Herein, we contribute two structurally different, but comparable copper borosulfates and explore the reasons for the different dimensionalities of the anionic substructure of borosulfates as well as the relationship between structure, stability, and acidity.

The copper borosulfate, Cu[B_2_(SO_4_)_4_], and the singly protonated species, Cu[B(SO_4_)_2_(HSO_4_)], were obtained under harsh conditions from a solution of fuming sulfuric and boric acid as colorless crystals (Figure S1 in the Supporting Information and the Experimental Section). Both compounds reveal Cu^II^ cations coordinated by six oxygen atoms, whereas a Jahn–Teller distortion is clear (Figures S2 and S3). These Cu^II^ cations are charge compensated by borosulfate anions. Cu[B_2_(SO_4_)_4_] exhibits a chain‐like anionic subunit according to the Niggli formula ${}_{\infty }^{1}{\left[B{\left({\mathrm{SO}}_{4}\right)}_{4/\;2}\right]}^{-}$ (Figures S4 and S5). In contrast to the published borosulfates *A*[B(SO_4_)_2_] (*A*=Ag^+^, Na^+^, K^+^, NH_4_^+^, H_3_O^+^),^2^ the chain‐like anions show both a twisted conformation of the B(SO_4_)_2_B substructures and a unique chair arrangement (Figure 1). Cu[B(SO_4_)_2_(HSO_4_)] exhibits separated dimeric complex anions [B_2_(SO_4_)_4_(HSO_4_)_2_]^4−^ (Figures S6 and S7) exclusively with a twisted conformation. It figuratively represents a cut‐out and selection of the twisted conformer from the anionic chain in Cu[B_2_(SO_4_)_4_] owing to protonation (Figure 2). With respect to the compounds *A*[B(SO_4_)_2_] (*A* = Ag^+^, Na^+^, K^+^, NH_4_^+^, H_3_O^+^),^2^ it could be thought that the dimensionality of the anionic substructure might depend on the charge of the counter cation, which is disproved by the herein presented Cu^II^ species.

**Figure 1:**
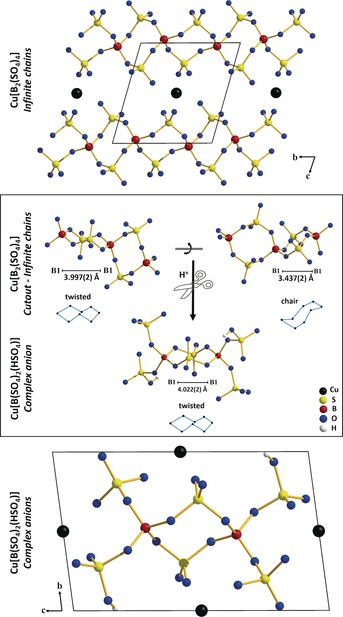
Top/Bottom: Crystal structures of Cu[B_2_(SO_4_)_4_] and Cu[B(SO_4_)_2_(HSO_4_)] in projection along the *a*‐axes. Middle: The twisted and the chair conformation of the B1(SO_4_)_2_B1 subunits in the anionic chain ${}_{\infty }^{1}{\left[B{\left({\mathrm{SO}}_{4}\right)}_{4/\;2}\right]}^{-}$ of Cu[B_2_(SO_4_)_4_] with a B1‐B1 distance of 3.997(2) Å in the twisted and a shorter B1‐B1 distance of 3.437(2) Å in the chair conformation as well as the protonated borosulfate anion [B_2_(SO_4_)_4_(HSO_4_)_2_]^4−^ in Cu[B(SO_4_)_2_(HSO_4_)] with a B1‐B1 distance of 4.022(2) Å and exclusively twisted conformation.

**Figure 2:**
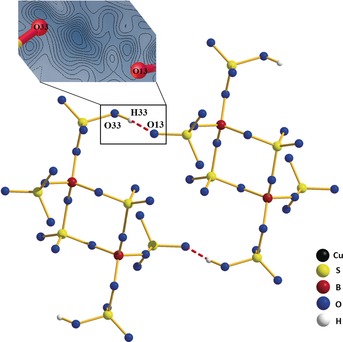
Hydrogen bonding between two [B_2_(SO_4_)_4_(SO_4_H)_2_]^4−^ anions in Cu[B(SO_4_)_2_(HSO_4_)]. Top‐left: Difference Fourier map of the strong hydrogen bond O13⋅⋅⋅H33⋅⋅⋅O33 (O33–H33 0.90(5) Å, O33–O13 1.459(1) Å).^3^

In fact, charge and size of the cations seem to be of minor importance for the dimensionality of the borosulfate anions, which is furthermore clearly emphasized by the herein presented simultaneous crystallization of Cu[B_2_(SO_4_)_4_] (anionic chain) and Cu[B(SO_4_)_2_(HSO_4_)] (separated complex anion; Experimental Section).

To further investigate the new borosulfates, magnetochemical measurements and density functional theory (DFT) calculations were performed. The temperature dependence of the magnetic susceptibility *χ* of Cu[B(SO_4_)_2_(HSO_4_)] was measured at an external field of 10 kOe (Figure 3, top). The inverse susceptibility *χ* ^−1^ is linear above 75 K and was fitted using the Curie–Weiss law (red line; Figure 3). The effective magnetic moment was calculated to *μ*_eff_=1.91(1) μ_B_, the Weiss constant is large (*θ*_P_=−140(1) K), pointing towards antiferromagnetic interactions in the paramagnetic regime, however, no intrinsic magnetic transition was observed down to low temperatures. The extracted magnetic moment is higher, when compared to a spin‐only high‐spin Cu^2+^ cation (d^9^, *μ*_calcd_=1.73 μ_B_) in a distorted octahedral coordination environment. This can be explained by additional contributions from orbital angular momentum and spin‐orbit coupling in Cu[B(SO_4_)_2_(HSO_4_)], similar to what has been observed for CuSO_4_⋅5 H_2_O.^4^ In general, the reported experimental effective magnetic moments for Cu^2+^ are often listed to be between 1.70 and 2.20 μ_B_.^5^ A small anomaly can be observed around 35 K, which, however, cannot be assigned to possible side products. The magnetization isotherms recorded at 10 and 50 K exhibit a linear trend, as expected for a paramagnetic material (Figure 3, bottom). The 3 K isotherm shows a curved trace with a saturation magnetization of *μ*_sat_=0.35(1) μ_B_ at 3 K and 80 kOe. Since the magnetic behavior of Cu[B(SO_4_)_2_(HSO_4_)] is now known, a sample containing both Cu[B(SO_4_)_2_(HSO_4_)] and Cu[B_2_(SO_4_)_4_] was measured to obtain information about the magnetic features of Cu[B_2_(SO_4_)_4_]. Again, no magnetic ordering was observed (Figure S11). Owing to the mixture, the calculation of the effective magnetic moment was not possible, however, it is undoubtedly clear that the Cu atoms are in the Cu^II^ oxidation state.

**Figure 3:**
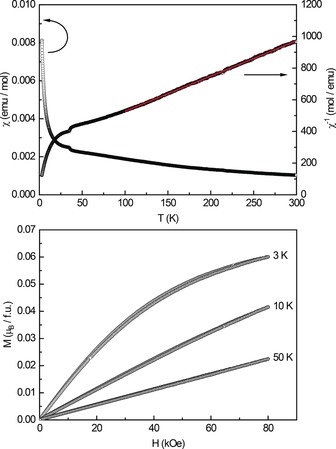
Top: Magnetic behavior of Cu[B(SO_4_)_2_(HSO_4_)]: magnetic susceptibility measured in ZFC mode at 10 kOe, the Curie–Weiss fit is depicted in red. Bottom: Magnetization isotherms recorded at 3, 10, and 50 K.

DFT calculations confirmed the experimentally determined X‐ray single‐crystal structures of Cu[B_2_(SO_4_)_4_] and Cu[B(SO_4_)_2_(HSO_4_)] which is the second protonated borosulfate after H[B(SO_4_)(S_2_O_7_)].2e Structural parameters are in good agreement with the experimental values (Table S10). Analysis of the Hirshfeld charges (Table S11) revealed positively charged Cu ions in both borosulfates. Sulfur, boron, and in the case of Cu[B(SO_4_)_2_(HSO_4_)], hydrogen atoms also carry positive charges, while all oxygen atoms are negatively charged. These results indicate the close structural relation of the two investigated borosulfates to silicates as previously found for CaB_2_S_4_O_16_.1c Despite these findings, we use the sum formula Cu[B_2_(SO_4_)_4_], particularly in comparison to Cu[B(SO_4_)_2_(HSO_4_)]. Hirshfeld spin density analysis (Table S12) shows that for both borosulfates the spin density is almost exclusively located on Cu, corroborating the d^9^ configuration of copper and the paramagnetic behavior.

To explore why also a chair conformation of the B(SO_4_)_2_B substructure is found in Cu[B_2_(SO_4_)_4_] and only a twisted conformation of the [B_2_(SO_4_)_4_(HSO_4_)_2_]^4−^ anion in Cu[B(SO_4_)_2_(HSO_4_)], a hypothetical chair conformation of the complex anion [B_2_(SO_4_)_4_(HSO_4_)_2_]^4−^ was constructed based on the B(SO_4_)_2_B substructure (Figure 4). The protonated, twisted conformation is by Δ*G*=25.5 kJ mol^−1^ more stable than the chair conformation. Upon deprotonation, the chair conformation is not stable and converts to a half‐twist (Figure 4), thus, this conformation can only be stabilized by means of charge delocalization in the extended complex anionic chain found for Cu[B_2_(SO_4_)_4_].

**Figure 4:**
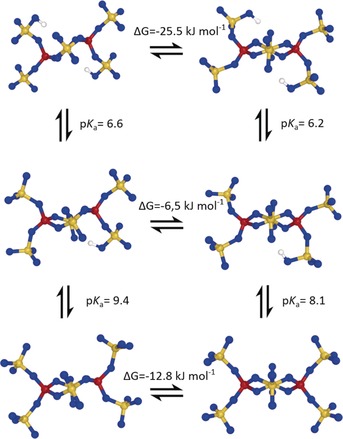
Calculated free‐energy differences Δ*G* of the complex anion [B_2_(SO_4_)_4_(HSO_4_)_2_]^4−^ between the hypothetical chair conformation (left) and the experimentally found twist conformation (right) as well as p*K*_a_ values for the deprotonation to [B_2_(SO_4_)_5_(HSO_4_)]^5−^ and to [B_2_(SO_4_)_6_]^6−^.

To assess the relative acidity of the [B_2_(SO_4_)_4_(HSO_4_)_2_]^4−^ anion, p*K*_a_ values were calculated (for details see Quantum chemical methodology in the Experimental Section) and compared to those of H_2_SO_4_ and Si(OH)_4_. For the two conformations, p*K*_a_ values of 6.6 (chair) and 6.2 (twist) were calculated for the first deprotonation and p*K*_a_ values of 9.4 (chair) and of 8.1 (twist), respectively, for the second deprotonation step (Table S15), showing little dependence on the conformation. In comparison, when calculated with the same methodology, a p*K*_a_ of −5.3 is found for the first deprotonation of H_2_SO_4_ and a p*K*_a_ of 17.5 for Si(OH)_4_. This emphasizes that the borosulfate anion [B_2_(SO_4_)_4_(HSO_4_)_2_]^4−^ is not a strong acid but behaves rather silicate‐like.

In summary, the new copper borosulfates Cu[B_2_(SO_4_)_4_] and Cu[B(SO_4_)_2_(HSO_4_)] illustrate that the dimensionality of the borosulfate anion depends on a variety of factors. Nevertheless, there is a strong relationship between dimensionality, stability, and properties, such as the p*K*_a_ value. In fact, the calculated p*K*_a_ values of the investigated borosulfates are more comparable to those of silicic acid, indicating once again the close relationship of borosulfates to silicates and thus the significance of this scarcely explored class of compounds.

## Experimental Section

***Caution***: Oleum is a strong oxidizer which needs careful handling. During and even after the reaction, the ampoule might be under remarkable pressure. It is mandatory to cool down the ampoule by liquid nitrogen prior to opening.

Synthesis of Cu[B_2_(SO_4_)_4_] and Cu[B(SO_4_)_2_(HSO_4_)]: Cu_2_O (0.52 mmol, Sigma–Aldrich, Darmstadt, Germany), H_3_BO_3_ (1.6 mmol, Carl Roth, Karlsruhe, Germany), and 1 mL oleum (20 % SO_3_, Sigma–Aldrich, Darmstadt, Germany) were loaded into a thick‐walled glass ampoule (*l*=300 mm, Ø=16 mm, thickness of the tube wall: 1.8 mm). The ampoule was torch sealed, placed into a tube resistance furnace and heated up to 453 K. The temperature was maintained for 48 hours and finally decreased to 298 K with a cooling rate of 0.1 K min^−1^. A large number of colorless crystals was obtained (Figure S1), and the yield was almost quantitative with respect to the initial copper oxide. Single‐crystal and powder X‐ray diffraction experiments revealed the copper borosulfates Cu[B_2_(SO_4_)_4_] and Cu[B_2_(SO_4_)_2_(HSO_4_)], simultaneously.

The bulk material was washed in a glove box with anhydrous hexane. After drying the sample under reduced pressure, a suitable sample for X‐ray powder diffraction was prepared under inert atmosphere. The obtained powder pattern (Figure S8) revealed predominantly reflections of Cu[B_2_(SO_4_)_2_(HSO_4_)]. Cu[B_2_(SO_4_)_4_] could not be re‐crystallized from the removed hexane. Multiple attempts to realize the phase‐pure synthesis of Cu[B_2_(SO_4_)_4_] were not successful, neither by substitution of the initial copper species, change of the SO_3_‐concentration nor by variation of the reaction temperature. However, we were able to increase the content of Cu[B_2_(SO_4_)_4_] up to an amount of 22(1)% (amounts from X‐ray powder diffraction) by tuning the cooling rate to 2.2 K min^−1^ and using a block‐shaped furnace.

Both types of crystals are very moisture sensitive and decompose immediately after exposure to air. Thus, they were handled under strictly inert conditions for further investigations.

*X‐ray crystallography*: The mother liquor was separated from the crystals via decantation. The ampoule was cooled with liquid nitrogen and several crystals (Figure S1) were transferred into inert oil directly after opening the ampoule. The remaining bulk material was transferred to a glovebox for further characterization. Under a polarization microscope, suitable crystals were prepared, mounted onto a glass needle (Ø=0.1 mm) and immediately placed into a stream of cold N_2_ (173(2) K) inside the diffractometer (Bruker D8 Quest κ, Bruker, Karlsruhe, Germany). After unit cell determination, the reflection intensities were collected. Cu[B_2_(SO_4_)_4_]: colorless block (0.10×0.05×0.015 mm), triclinic, $P\bar{1}$ , *Z*=1, *a*=5.2470(3) Å, *b*=7.1371(3) Å, *c*=7.9222(5) Å, *α*=73.814(3)°, *β*=70.692(2)°, *γ*=86.642(2)°, *V*=268.71(3) Å^3^, *ρ*=2.90 g cm^−3^, 2*θ*_max._=75.7°, *λ*(Mo‐*K*_*α*_)=71.073 pm, 18 180 reflections, 2895 unique reflections (*R*_int_=0.0463), multi‐scan absorption correction (*μ*=29.2 cm^−1^, min./max. transmission=0.701/0.747, program SADABS‐2014/5: Bruker, Germany 2014), structure solution by Direct Methods, full‐matrix‐least‐squares refinement (107 parameters) on |*F* ^2^|, anisotropic refinement for all atoms (programs SHELXS and SHELXL: G. M. Sheldrick, *Acta Crystallogr*. **2008**, *A64*, 112—122, Germany 2008), *R*1=0.0286, *wR*2=0.0588 for 2388 reflections with *I*>2*σ*(*I*) and *R1=*0.0432, *wR2=*0.0632 for all 2895 reflections, max./min. residual electron density=0.67/−0.84 e^−^ Å^−3^. Further details on the crystal structure investigations may be obtained from the Fachinformationszentrum Karlsruhe, 76344 Eggenstein‐Leopoldshafen, Germany (fax: (+49) 7247‐808‐666; e‐mail: crysdata@fiz‐karlsruhe.de), on quoting the depository number CSD‐432821 and are listed in Table S1.

Cu[B(SO_4_)_2_(HSO_4_)]: colorless plates (0.11×0.095×0.09 mm), triclinic, $P\bar{1}$ , *Z*=1, *a*=5.3096(7) Å, *b*=7.0752(4) Å, *c*=11.2977(6) Å, *α*=81.154(1)°, *β*=80.302(2)°, *γ*=80.897(4)°, *V*=409.54(4) Å^3^, *ρ*=2.95 g cm^−3^, 2*θ*_max._=75.8°, *λ*(Mo‐*K*_*α*_)=71.073 pm, 22 044 reflections, 4407 unique reflections (*R*_int_=0.0212), multi‐scan absorption correction (*μ*=35.0 cm^−1^, min./max. transmission=0.712/0.747, program SADABS‐2014/5: Bruker, Germany 2014), structure solution by Direct Methods, full‐matrix‐least‐squares refinement (162 parameters) on |*F* ^2^|, anisotropic refinement for all non‐hydrogen atoms, hydrogen atom located in the difference Fourier map and freely refined (programs SHELXS and SHELXL: G. M. Sheldrick, *Acta Crystallogr*. **2008**, *A64*, 112—122, Germany 2008), *R*1=0.0262, *wR*2=0.0699 for 4058 reflections with *I*>2*σ*(*I*) and *R*1=0.0302, *wR*2=0.0721 for all 4407 reflections, max./min. residual electron density=0.65/−1.96 e^−^ Å^−3^. Further details on the crystal structure investigations may be obtained from the Fachinformationszentrum Karlsruhe, 76344 Eggenstein‐Leopoldshafen, Germany (fax: (+49) 7247‐808‐666; e‐mail: crysdata@fiz‐karlsruhe.de), on quoting the depository number CSD‐432820 and are listed in Table S1.

*X‐ray powder diffraction*: The measurement was carried out with a Stoe Stadi P powder diffractometer in transmission geometry. The flat sample was irradiated with Ge(111)‐monochromatized Mo‐*K*_*α*1_‐radiation (*λ*=0.7093 Å), which was detected by means of a Dectris Mythen 1 K detector. Rietveld refinement was accomplished using TOPAS 4.2 (Bruker, Germany, 2009).

*Magnetochemical investigations of Cu[B_2_(SO_4_)_2_(HSO_4_)]*: The sample was packed into a PE capsule in an argon filled glove box and attached to the sample holder rod of a Vibrating Sample Magnetometer unit (VSM) for measuring the magnetization *M*(*T*) in a Quantum Design Physical‐Property‐Measurement‐System (PPMS). The sample was investigated in the temperature range of 2.5–300 K with magnetic flux densities up to 80 kOe.

*Quantum chemical methodology*: The experimentally obtained single‐crystal structures were subject to structure optimization using DFT with periodic boundary conditions as implemented in CRYSTAL14.^6^ Spin‐unrestricted open‐shell calculations were performed with two different density functionals, the PBESOL^7^ and the hybrid range‐separated HSESOL^8^ density functional. All‐electron basis sets were used for Cu,^9^ O,^10^ and B,^11^ and effective core potentials for S.^12^ To further analyze the electronic structure, an iterative Hirshfeld population analysis was performed as implemented in CRYSTAL17,^13^ where the partial charge at each atom was calculated as the difference of the total electronic density and a promolecular atomic density.

To compare the stability of the complex anion [B_2_(SO_4_)_4_(HSO_4_)_2_]^4−^ in its experimentally observed twisted to a hypothetical chair conformation, obtained as a cut out of the B(SO_4_)_2_B subunits and subsequent protonation, relative electronic energies were calculated. Zero‐point energies and thermal corrections were added to yield relative Gibbs free energies at standard conditions.

p*K*_a_ values of the complex anion [B_2_(SO_4_)_4_(HSO_4_)_2_]^4−^ in twisted and chair conformation were obtained in aqueous solution (modelled as implicit solvent) according to the thermodynamic cycle depicted in Scheme S1 from the Gibb's free energy of solvation. The gas phase free energy of H^+^ is set to *G*_(g)_(H^+^)=−26.32 kJ mol^−1^,^14^ whereas the solvent free energy is Δ*G*_(solv)_(H^+^)=−1087.00 kJ mol^−1^ (Scheme S1).^15^ Calculations of molecular structures were performed with the quantum chemical suite TURBOMOLE^16^ employing the BP86 density functional,^17^ together with the resolution of identity technique^18^ and the def2‐TZVP basis set of triple‐zeta quality.^19^ To model the implicit solvent, the conductor‐like screening model (COSMO) was used as implemented in TURBOMOLE with a dielectric constant of *ϵ*=78.5. All structures were visualized with PyMOL.^20^

## Conflict of interest

The authors declare no conflict of interest.

## Supporting information

As a service to our authors and readers, this journal provides supporting information supplied by the authors. Such materials are peer reviewed and may be re‐organized for online delivery, but are not copy‐edited or typeset. Technical support issues arising from supporting information (other than missing files) should be addressed to the authors.

Supplementary
