# Supplementary material for: Cu[B2(SO4)4] and Cu[B(SO4)2(HSO4)]—Two Silicate Analogue Borosulfates Differing in their Dimensionality: A Comparative Study of Stability and Acidity
Source: Angew Chem Int Ed Engl. 2018 May 16;57(30):9548–52. doi: 10.1002/anie.201803395 (PMC6099308; doi:10.1002/anie.201803395)
Supplement: Supplementary file 1 — Supplementary [file ANIE-57-9548-s001.pdf]

Angewandte Chemie
Eine Zeitschrift der Gesellschaft Deutscher Chemiker

Supporting Information

$Cu[B_2(SO_4)_4]$ and $Cu[B(SO_4)_2(HSO_4)]$—Two Silicate Analogue Borosulfates Differing in their Dimensionality: A Comparative Study of Stability and Acidity

Jörn Bruns, Maren Podewitz, Klaus R. Liedl, Oliver Janka, Rainer Pöttgen, and Hubert Huppertz**

anie_201803395_sm_miscellaneous_information.pdf

Supporting information

Crystallography

Empirical formula	$CuB_2S_4O_{16}$	$CuBS_3O_{12}H$
Formula weight	469.40	363.54
Temperature/ K	173(2)	173(2)
Crystal system	triclinic	triclinic
Space group	$P\bar{1}$ (no. 2)	$P\bar{1}$ (no. 2)
a/ Å	5.2470(3)	5.3096(3)
b/ Å	7.1371(3)	7.0752(4)
c/ Å	7.9222(5)	11.2977(6)
α/ °	73.814(3)	81.154(1)
β/ °	70.692(2)	80.302(2)
γ/ °	86.642(2)	80.897(4)
Volume/ Å^3	268.71(3)	409.54(4)
Z	1	2
ρ_{calc}/ gcm^{-3}	2.90	2.95
Absorption coefficient/ cm^{-1}	29	35
F(000)	231	358
Crystal size/ mm^3	0.1 × 0.05 × 0.015	0.11 × 0.095 × 0.09
2ϑ range for data collection	5.7 to 75.7°	5.9 to 75.8°
Index ranges	±9, ±12, ±13	±9, ±12, ±19
Reflections collected	18180	22044
Independent reflections	2895 [R_{int} = 0.0463]	4407 [R_{int} = 0.0212]
Completeness to theta	1.00	0.999
Absorption correction	multi-scan	multi-scan
Refinement method	Full-matrix least-squares on F^2	Full-matrix least-squares on F^2
Data / parameters	2895 / 107	4407 / 162
Goodness-of-fit on F^2	1.049	1.042
Final R indexes [$I \geq 2\sigma(I)$]	R_1 = 0.0286, wR_2 = 0.0588	R_1 = 0.0262, wR_2 = 0.0699
Final R indexes [all data]	R_1 = 0.0432, wR_2 = 0.0632	R_1 = 0.0302, wR_2 = 0.0721
Largest diff. peak/hole/ e$^-$/Å^{-3}	0.67 / –0.84	0.65 / –1.96

CSD number	432821	432820

Table S1. Crystal data and structure refinement for $Cu[B_2(SO_4)_4]$ and $Cu[B(SO_4)_2(HSO_4)]$.

Table S2. Fractional atomic coordinates (×10^4) and equivalent isotropic displacement parameters (Å^2×10^3) for $Cu[B_2(SO_4)_4]$. U_{eq} is defined as 1/3 of the trace of the orthogonalised U_{ij} tensor.

Atom	*x*	*y*	*z*	*U(eq)*
Cu1	5000	5000	5000	5.99(6)
S2	2137.3(7)	5073.3(5)	2051.1(4)	5.00(6)
S1	5925.0(7)	9273.2(5)	2493.8(5)	5.98(6)
O21	4027(2)	4280(2)	3032(2)	8.2(2)
O23	2102(2)	3779(2)	825(2)	7.6(2)
O121	3212(2)	7051(2)	669(2)	7.4(2)
O11	4253(2)	7687(2)	3973(2)	10.0(2)
O13	3850(2)	10738(2)	2056(2)	9.6(2)
O111	7219(2)	8579(2)	751(2)	10.0(2)
O22	-546(2)	5325(2)	3123(2)	10.5(2)
O12	7961(2)	10102(2)	2880(2)	15.5(2)
B1	6035(3)	7766(2)	-343(2)	7.3(2)

Table S3. Anisotropic displacement parameters (Å^2×10^3) for $Cu[B_2(SO_4)_4]$. The anisotropic displacement factor exponent takes the form: -$2\pi^2[h^2a^{*2}U_{11}+...+2hka\times b\times U_{12}]$

Atom	U_{11}	U_{22}	U_{33}	U_{23}	U_{13}	U_{12}
Cu1	8.8(1)	4.5(1)	4.8(1)	-0.44(7)	-3.26(8)	0.93(7)
S2	5.2(1)	4.9(1)	4.3(1)	-0.96(9)	-1.0(1)	0.54(9)
S1	7.8(1)	5.4(1)	5.3(1)	-1.5(1)	-3.0(1)	1.0(1)
O21	11.6(4)	7.4(4)	7.6(4)	-1.9(3)	-6.2(3)	2.4(3)
O23	10.2(4)	6.3(4)	8.9(4)	-4.0(3)	-5.2(3)	2.5(3)
O121	7.2(4)	5.3(4)	7.8(4)	0.7(3)	-2.1(3)	-0.4(3)
O11	12.8(5)	6.5(4)	7.1(4)	0.9(3)	-1.4(4)	1.7(3)
O13	10.9(4)	7.4(4)	6.9(4)	1.2(3)	-1.6(3)	3.5(3)
O111	9.9(4)	9.6(4)	7.6(4)	-4.7(3)	-1.3(3)	0.1(3)
O22	7.1(4)	12.2(5)	8.8(4)	-2.4(4)	1.3(3)	1.6(3)

O12	13.1(5)	21.9(6)	15.6(5)	-8.5(4)	-6.6(4)	-2.7(4)
B1	8.4(6)	5.9(6)	6.9(6)	-1.2(5)	-2.3(5)	0.9(5)

Table S4. Experimental bond lengths for $Cu[B_2(SO_4)_4]$.

Atom	Atom	length/ Å	Atom	Atom	length/ Å
Cu1	O21	1.9900(1)	S1	O13	1.521(1)
Cu1	O11^1	1.935(1)	S1	O12	1.405 (1)
Cu1	O22^2	2.302(1)	S1	O12	1.405(1)
S2	O21	1.451(1)	B1^3	O23	1.476(2)
S2	O23	1.5200(1)	B1	O121	1.474(2)
S2	O121	1.527(1)	B1^4	O13	1.460(2)
S2	O22	1.414(1)	B1	O111	1.470(2)
S1	O11	1.457(1)	B1	O23^3	1.476(2)
S1	O111	1.528(1)	B1	O13^4	1.460(2)

^{1}1-*x*,1-*y*,1-*z*; ^{2}1+*x*,+*y*,+*z*; ^{3}1-*x*,1-*y*,-*z*; ^{4}1-*x*,2-*y*,-*z*

Table S5. Experimental bond angles for $Cu[B_2(SO_4)_4]$.

Atom	Atom	Atom	Angle/°	Atom	Atom	Atom	Angle/°
O21^1	Cu1	O21	180	O13	S1	O111	106.01(6)
O21^1	Cu1	O22^2	91.09(4)	O12	S1	O11	116.94(7)
O21	Cu1	O22^2	88.91(4)	O12	S1	O13	112.67(7)
O21	Cu1	O22^3	91.09(4)	O12	S1	O111	108.63(7)
O11^1	Cu1	O21^1	89.11(4)	S2	O21	Cu1	132.41(7)
O11	Cu1	O21^1	90.89(4)	B1^4	O23	S2	128.79(9)
O11	Cu1	O11^1	180	B1	O121	S2	128.97(9)

O11	Cu1	O22[2]	93.69(4)	S1	O11	Cu1	132.00(7)
O11[1]	Cu1	O22[2]	86.31(4)	B1[5]	O13	S1	130.4(1)
O22[3]	Cu1	O22[2]	180	B1	O111	S1	131.6(1)
O21	S2	O23	107.76(6)	S2	O22	Cu1[6]	167.35(7)
O21	S2	O121	110.15(6)	O121	B1	O23[4]	113.6(1)
O23	S2	O121	103.89(6)	O13[5]	B1	O23[4]	108.6(1)
O22	S2	O21	117.86(7)	O13[5]	B1	O121	109.1(1)
O22	S2	O23	109.27(6)	O13[5]	B1	O111	109.1(1)
O22	S2	O121	107.03(6)	O111	B1	O23[4]	103.3(1)
O11	S1	O13	102.70(6)	O111	B1	O121	113.0(1)
O11	S1	O111	109.28(6)				

[1]1-*x*,1-*y*,1-*z*; [2]1+*x*,+*y*,+*z*; [3]-*x*,1-*y*,1-*z*; [4]1-*x*,1-*y*,-*z*; [5]1-*x*,2-*y*,-*z*; [6]-1+*x*,+*y*,+*z*

Table S6. Fractional atomic coordinates (×10^4) and equivalent isotropic displacement parameters (Å^2×10^3) for Cu[B(SO$_4$)$_2$(HSO$_4$)]. U_{eq} is defined as 1/3 of the trace of the orthogonalised U_{ij} tensor.

Atom	*x*	*y*	*z*	*U(eq)*
Cu1	0	5000	10000	6.45(5)
Cu2	10000	10000	5000	7.51(5)
S2	6762.7(6)	7007.8(4)	4554.3(3)	4.69(5)
S1	1095.2(6)	8013.5(4)	7646.7(3)	5.50(6)
S3	5361.0(6)	2758.8(4)	8792.4(3)	5.75(6)
O31	2611(2)	3372(2)	9050(1)	9.2(2)
O122	2819(2)	4363(2)	6403.0(9)	7.4(2)
O13	1552(2)	9721(2)	8077(1)	10.7(2)
O12	-158(2)	8398(2)	6557.7(9)	8.9(2)
O121	6914(2)	5661(2)	5730.3(9)	7.4(2)
O22	4352(2)	8224(2)	4562(1)	9.6(2)
O11	-438(2)	6762(2)	8532.9(9)	9.0 (2)
O111	3834(2)	6914(2)	7334(1)	8.2(2)
O21	9067(2)	7926(2)	4303.1(9)	7.9(2)
O131	6399(2)	3808(2)	7568.0(9)	8.8(2)

O32	6922(2)	2981(2)	9655(1)	12.5(2)
O33	5706(2)	646(2)	8604(1)	12.2(2)
B1	4893(3)	5212(2)	6763(1)	5.9(2)

Table S7. Anisotropic displacement parameters (Å^2×10^3) for Cu[B(SO_4)$_2$(HSO_4)]. The anisotropic displacement factor exponent takes the form: $-2\pi^2[h^2a^{*2}U_{11}+...+2hka\times b\times U_{12}]$

Atom	U_{11}	U_{22}	U_{33}	U_{23}	U_{13}	U_{12}
Cu1	6.91(9)	7.02(9)	3.76(8)	0.80(7)	0.61(7)	1.09(7)
Cu2	10.4(1)	6.91(9)	5.98(9)	0.53(7)	-2.52(7)	-3.40(7)
S2	5.2(1)	4.8(1)	4.3(1)	-0.67(8)	-0.46(8)	-1.49(8)
S1	7.0(1)	4.7(1)	4.7(1)	-0.55(8)	-0.62(9)	-1.03(9)
S3	6.8(1)	5.9(1)	4.5(1)	-0.03(8)	-1.41(9)	-0.64(9)
O31	8.1(4)	9.7(4)	8.3(4)	-1.5(3)	1.7(3)	0.1(3)
O122	7.3(4)	8.9(4)	7.2(4)	-4.4(3)	0.2(3)	-2.7(3)
O13	12.2(4)	7.5(4)	14.1(4)	-5.1(3)	-4.2(3)	-0.6(3)
O12	11.8(4)	9.5(4)	5.9(4)	2.7(3)	-4.0(3)	-3.7(3)
O121	7.1(4)	8.8(4)	5.2(3)	1.9(3)	0.3(3)	-1.5(3)
O22	8.4(4)	9.0(4)	10.9(4)	-1.5(3)	-2.1(3)	1.6(3)
O11	8.6(4)	10.5(4)	6.4(4)	3.0(3)	-0.1(3)	-1.4(3)
O111	6.4(4)	8.4(4)	10.3(4)	-4.8(3)	-0.1(3)	-0.7(3)
O21	8.3(4)	8.5(4)	7.5(4)	-0.7(3)	0.0(3)	-5.1(3)

O131	7.8(4)	11.7(4)	5.2(3)	2.7(3)	-0.3(3)	-0.1(3)
O32	14.8(5)	16.7(5)	8.1(4)	0.1(3)	-5.8(3)	-5.7(4)
O33	10.9(4)	6.0(4)	19.6(5)	-2.8(3)	-2.2(4)	0.1(3)
B1	6.5(5)	6.8(5)	4.5(5)	-0.9(4)	-0.2(4)	-1.4(4)

Table S8. Experimental bond lengths for $Cu[B(SO_4)_2(HSO_4)]$.

Atom	Atom	length/ Å	Atom	Atom	length/ Å
Cu1	O31[1]	1.947(1)	S1	O11	1.459(1)
Cu1	O11[1]	1.939(1)	S1	O111	1.548(1)
Cu1	O32	2.447(1)	S3	O31	1.449(1)
Cu2	O12[2]	1.938(1)	S3	O131	1.525(1)
Cu2	O21[3]	1.933(1)	S3	O32	1.422(1)
Cu2	O22	2.460(1)	S3	O33	1.521(1)
S2	O122[4]	1.528(1)	O122	S2[4]	1.528(1)
S2	O121	1.517(1)	O122	B1	1.475(2)
S2	O22	1.425(1)	O121	B1	1.479(2)
S2	O21	1.441(1)	O111	B1	1.444(2)
S1	O13	1.438(1)	O131	B1	1.473(2)
S1	O12	1.465(1)	O33	H33	0.90(5)

[1]-*x*,1-*y*,2-*z*; [2]1-*x*,2-*y*,1-*z*; [3]2-*x*,2-*y*,1-*z*; [4]1-*x*,1-*y*1-*z*

Table S9. Experimental bond angles for $Cu[B(SO_4)_2(HSO_4)]$.

Atom	Atom	Atom	Angle/°	Atom	Atom	Atom	Angle/°
O31[1]	Cu1	O31	180	O31	S3	O131	108.76(6)
O11	Cu1	O31[1]	91.20(5)	O31	S3	O33	106.90(7)
O11	Cu1	O31	88.80(5)	O32	S3	O31	117.90(7)
O11	Cu1	O11[1]	180	O32	S3	O131	108.51(7)
O12[2]	Cu2	O12[3]	180	O32	S3	O33	109.19(7)
O21[4]	Cu2	O12[2]	90.50(4)	O33	S3	O131	104.81(7)

O21[4]	Cu2	O12[3]	89.50(4)	S3	O31	Cu1	143.64(7)
O21[4]	Cu2	O21	180	B1	O122	S2[4]	124.37(9)
O121	S2	O122[4]	103.61(6)	S1	O12	Cu2[5]	140.58(7)
O22	S2	O122[4]	111.94(6)	B1	O121	S2	130.74(9)
O22	S2	O121	112.28(6)	S1	O11	Cu1	137.81(7)
O22	S2	O21	117.50(7)	B1	O111	S1	135.22(9)
O21	S2	O122[4]	103.54(6)	S2	O21	Cu2	130.91(7)
O21	S2	O121	106.73(6)	B1	O131	S3	126.45(9)
O13	S1	O12	114.14(6)	O122	B1	O121	112.2(1)
O13	S1	O11	114.66(7)	O111	B1	O122	110.3(1)
O13	S1	O111	103.76(6)	O111	B1	O121	110.7(1)
O12	S1	O111	108.75(6)	O111	B1	O131	110.6(1)
O11	S1	O12	106.48(6)	O131	B1	O122	111.7(1)
O11	S1	O111	108.86(6)	O131	B1	O121	101.0(1)

[1]-*x*,1-*y*,2-*z*; [2]1+*x*,+*y*,+*z*; [3]1-*x*,2-*y*,1-*z*; [4]1-*x*,1-*y*,1-*z*; [5]-1+*x*,+*y*,+*z*

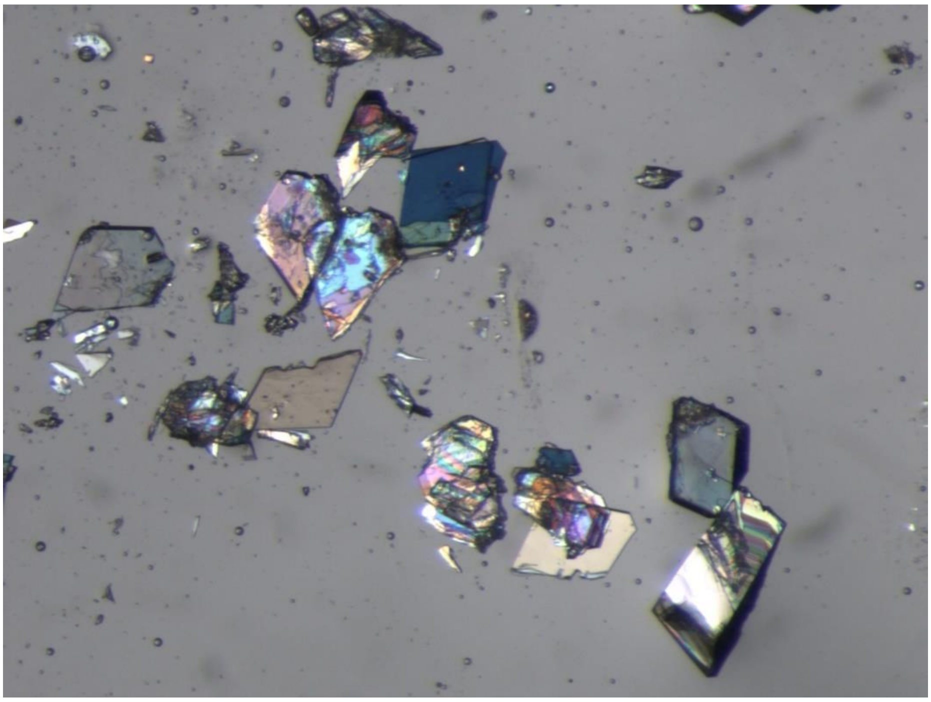

Figure S1. Single crystals of Cu[$B_2(SO_4)_4$] and Cu[$B(SO_4)_2(HSO_4)$] under a polarisation microscope. The picture is taken through the walls of a closed ampoule with the crystals being surrounded by oleum.

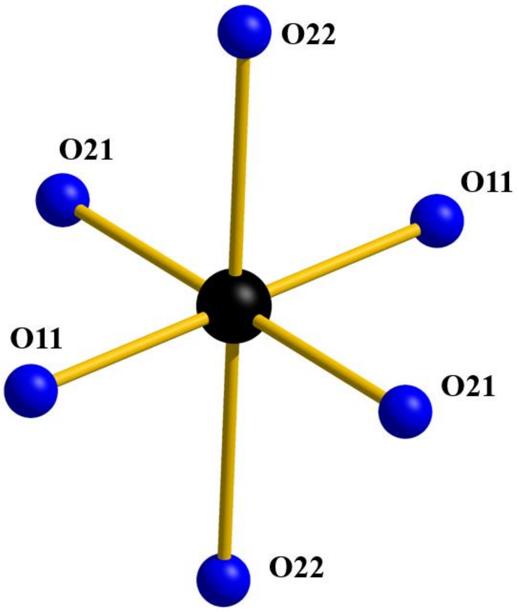

Figure S2. Octahedral oxygen coordination around Cu(II) cations in Cu[$B_2(SO_4)_4$], achieved by the coordination of six terminal oxygen atoms of the (SO_4) tetrahedra. Respectively, three of the six oxygen atoms are pairwise symmetrically equivalent (O11, O21 and O22), with the bond Cu1-O22 elongated to 2.302(1) Å compared to Cu1-O11 with 1.935(1) and 1.990(1) Å due to Jahn-Teller distortion.

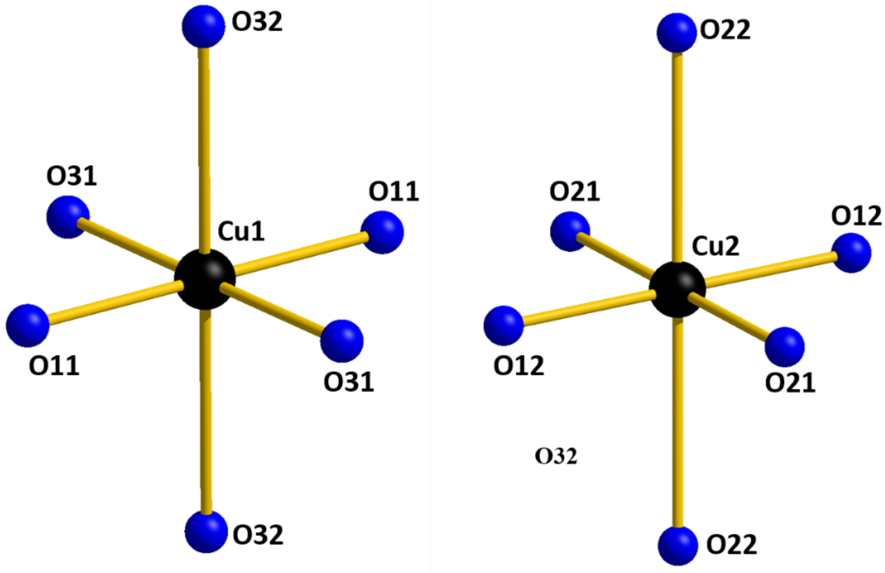

Figure S3. Octahedral oxygen coordination around two crystallographically independent Cu(II) cations in Cu[$B(SO_4)_2(HSO_4)$], achieved by the coordination of six terminal oxygen atoms of the (SO_4) tetrahedra. Respectively, three of the six oxygen atoms are pairwise symmetrically equivalent (O11, O31 and O32 for Cu1 and O12, O21 and O22 for Cu2). Respectively, the two axial bonds are elongated to 2.447(1) for Cu1-O32 and 2.460(1) for Cu2-O22 due to Jahn-Teller distortion. Further bond lengths [Å]: Cu1-O11 1.939(1), Cu1-O31 1.947(1), Cu2-O12 1.938(1), Cu2-O21 1.933(1).

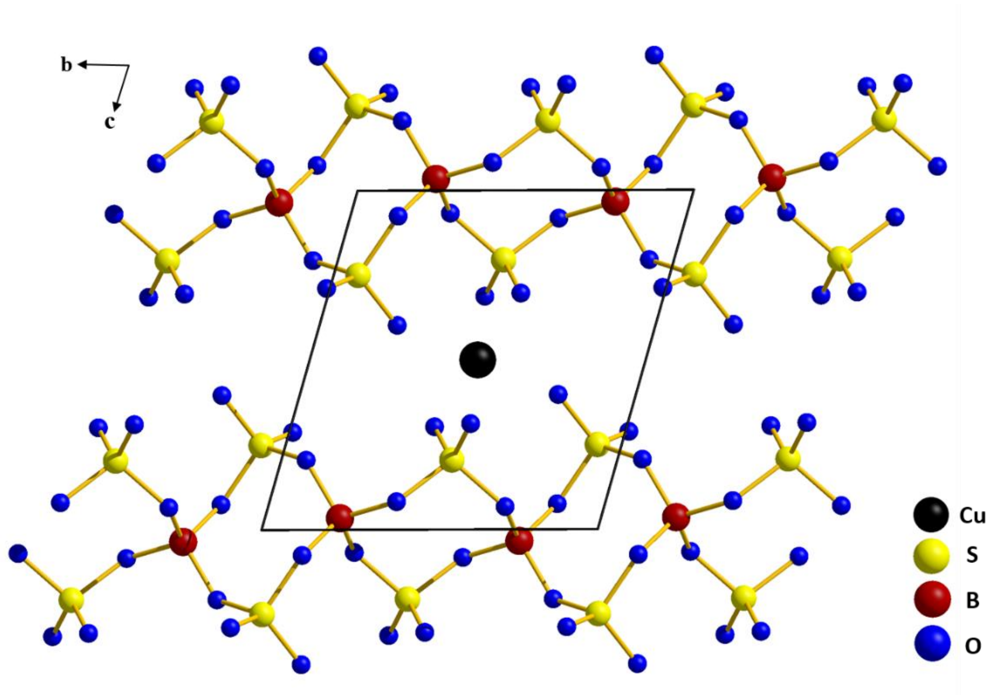

Figure S4. Crystal structure of Cu[$B_2(SO_4)_4$] in projection along [$\bar{1}00$]. Infinite anionic chains according to $^{1}_{\infty}[B(SO_4)_{4/2}]^{-}$ running parallel to the crystallographic *b*-axis. The chains are separated by Cu(II) cations.

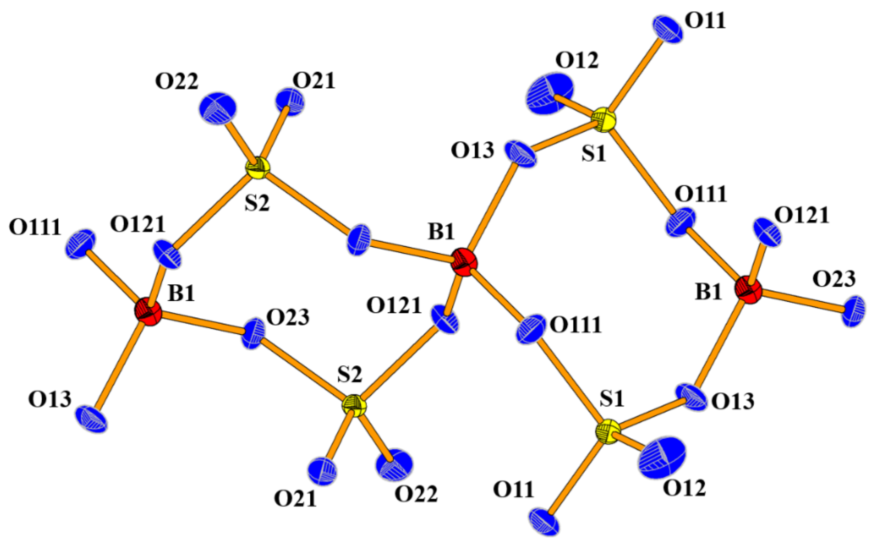

Figure S5. Connection of the (BO_4) and (SO_4) tetrahedra within the $^{1}_{\infty}[B(SO_4)_{4/2}]^{-}$ anions of Cu[$B_2(SO_4)_4$]. The displacement ellipsoids are set on 50 % probability level, the labelling is in accordance with the text and the tables in the supplement. Selected bond lengths [Å]: S1-O11 1.457(1), S1-O12 1.405(1), S1-O13 1.521(1), S1-O111 1.528(1), B1-O13 1.460(2), B1-O23 1.476(2), B1-O111 1.470(2), B1-O121 1.474(2).

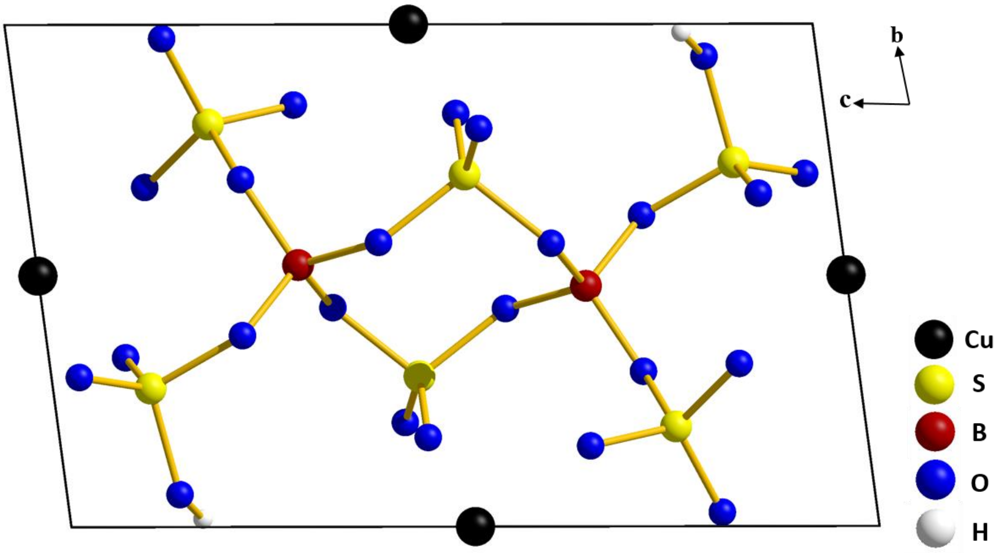

Figure S6. Crystal structure of Cu[B(SO$_4$)$_2$(HSO$_4$)] in projection along [$\bar{1}$00]. Complex oxoanions [B$_2$(SO$_4$)$_4$(SO$_4$H)$_2$]$^{4-}$ are separated by Cu(II) cations.

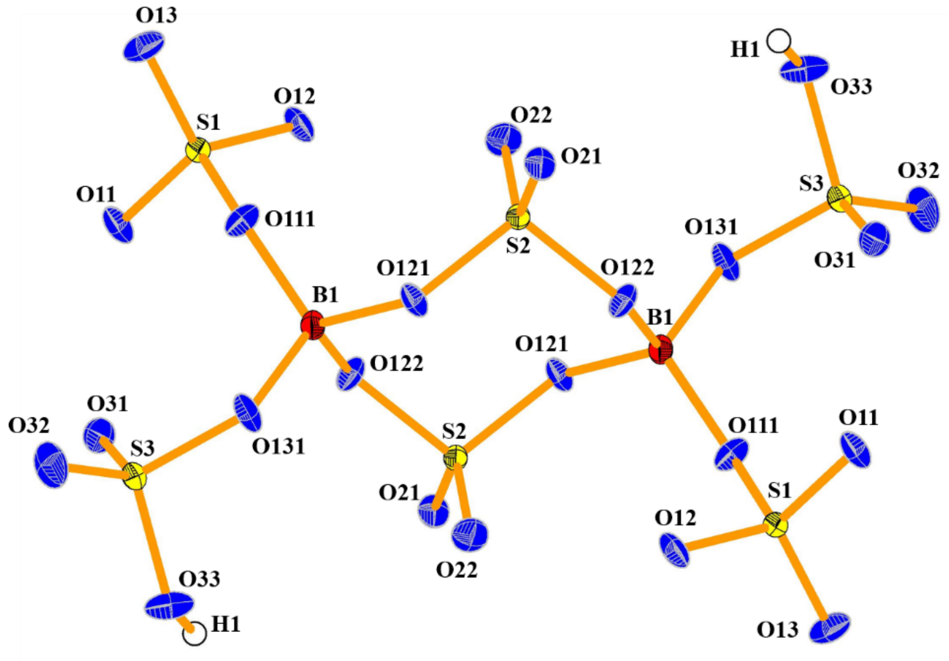

Figure S7. Connection of the (BO$_4$) and (SO$_4$) tetrahedra within the complex oxoanion [B$_2$(SO$_4$)$_4$(SO$_4$H)$_2$]$^{4-}$ of Cu[B(SO$_4$)$_2$(HSO$_4$)]. The displacement ellipsoids are set on 50 % probability level, the labelling is in accordance with the text and the tables in the supplement. Selected bond lengths [Å]: S1-O11 1.459(1), S1-O12 1.465(1), S1-O13 1.438(1), S1-O111 1.548(1), S2-O21 1.441(1), S2-O22 1.425(1), S2-O121 1.517(1), S2-O122 1.528(1), S3-

O31 1.449(1), S3-O32 1.422(1), S3-O33 1.521(1), S3-O131 1.525(1), B1-O111 1.444(2), B1-O121 1.479(2), B1-O122 1.475(2), B1-O131 1.473(2).

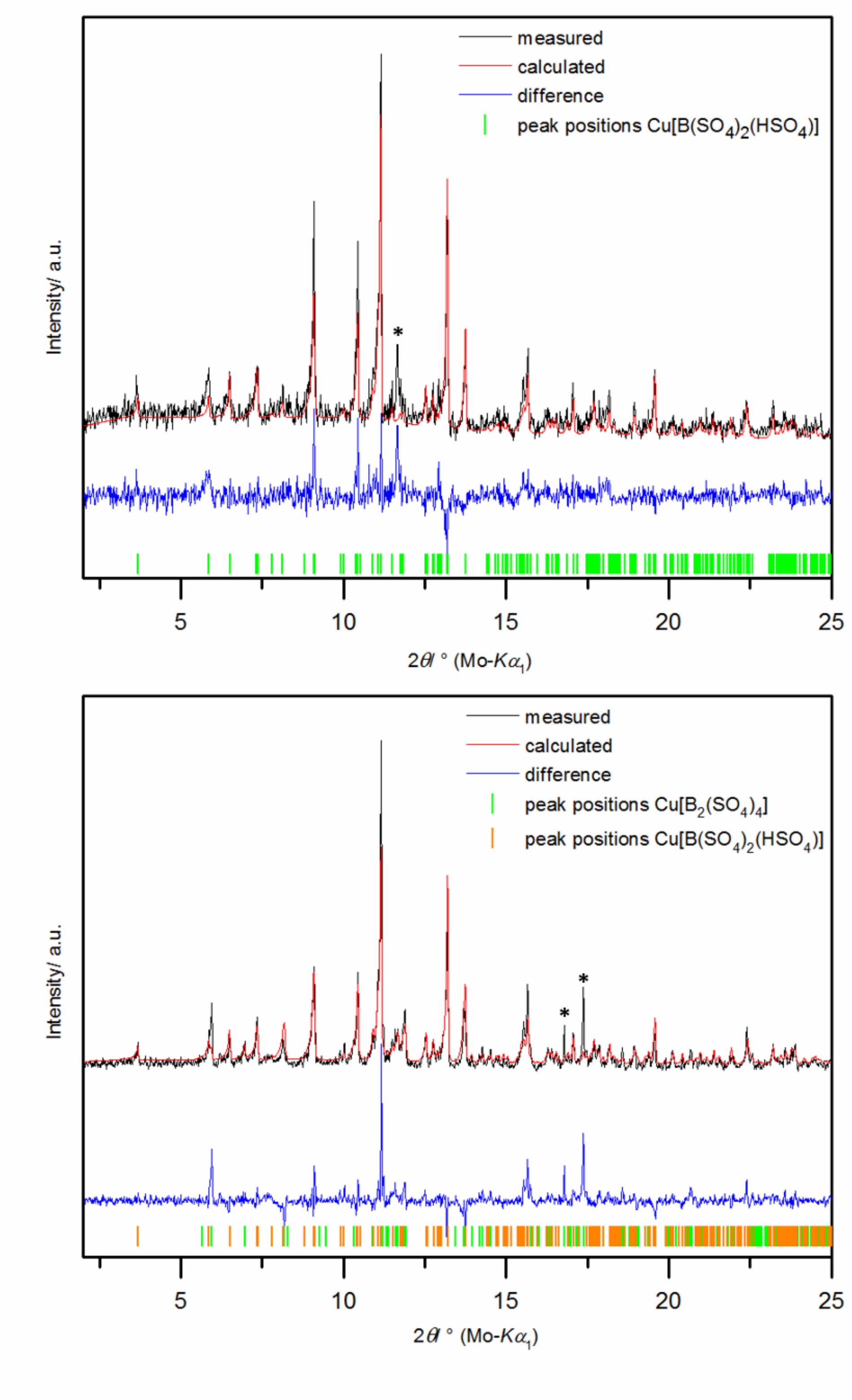

Figure S8. Top: Rietveld plot of $Cu[B(SO_4)_2(HSO_4)]$; Bottom: Rietveld plot of the mixture of $Cu[B_2(SO_4)_4]$ and $Cu[B(SO_4)_2(HSO_4)]$ with a ratio of 22(1)% : 78(1)%. Differing intensities might result from preferred orientation of the crystallites (platelet-like shape; see Figure S1). Reflections marked with an asterisk could not be further assigned. This impurity phase might account for the anomaly observed in the magnetochemical measurements.

Quantum Chemical Investigations

Calculation of Vibrational Spectra

Experimental vibrational spectra suffer either from small amounts of adhesive acid for the untreated sample, or from luminescent effects caused by adhesive hexane due to the washing process. Furthermore, $Cu[B_2(SO_4)_4]$ can only be obtained accompanied by $Cu[B(SO_4)_2(HSO_4)]$. Thus, we decided to calculate the spectra for a best possible characterization of each individual compound.

Although the structures of $Cu[B_2(SO_4)_4]$ and $Cu[B(SO_4)_2(HSO_4)]$ were optimized with both density functionals, PBESOL and HSESOL, for the subsequent frequency calculation only the HSESOL functional was employed, which is known to yield more accurate spectra [1].

IR and Raman frequencies were calculated within the harmonic approximation. Obtained normal modes were analyzed by the CRYSTAL's vibrational analysis tool and visual inspection and classified as stretching or bending modes (Table S13 and S14). To visualize the spectra, calculated IR intensities were fitted with Lorentzian functions using *J-ICE* (Figure S9 and S10) [2].

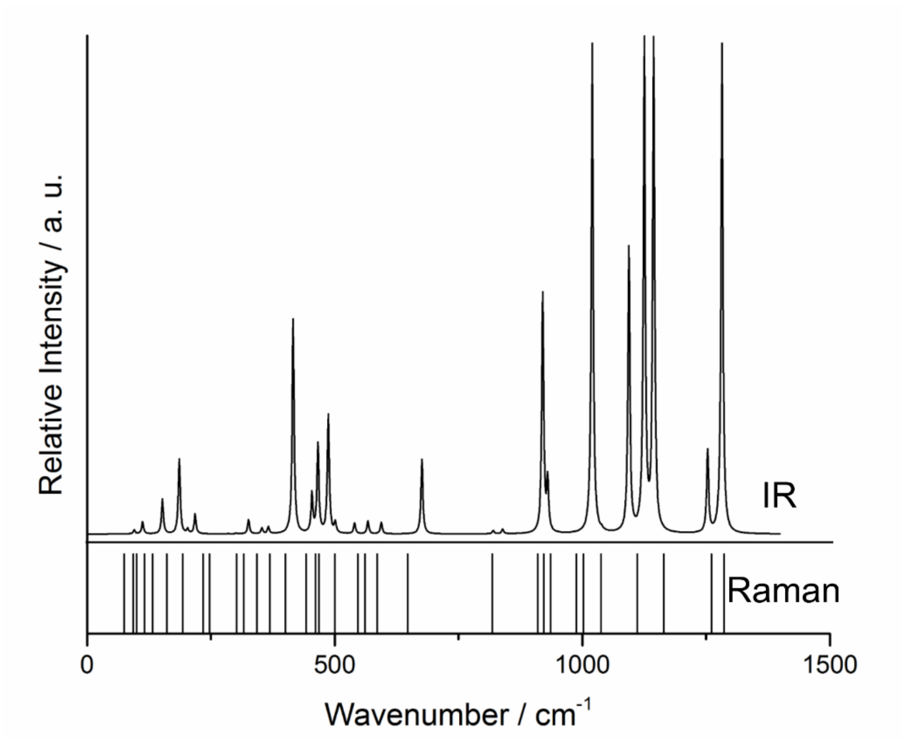

Figure S9. Calculated IR and Raman spectrum of $Cu[B_2(SO_4)_4]$. The highest intensity was arbitrarily set to 100% and all other intensities were scaled accordingly. Lorentzian line shape profiles were used to generate the spectra using *J-ICE* [2].

Calculated IR and Raman spectra of $Cu[B_2(SO_4)_4]$ revealed 66 vibrational modes. 33 of these modes are IR active and 33 are Raman active. The symmetry representation analysis of the Γ-point showed that 33 modes have A_g symmetry and 33 A_u symmetry. Calculated vibrational IR active modes were found between 95 and 1282 cm^{-1} as displayed in Figure S9, where a convoluted spectrum is displayed with Lorentzian shape line broadening. A complete assignment of modes is listed in Table S13. Stretching modes were found at larger wavenumbers, whereas bending modes predominate at smaller wavenumbers, thus lower energies. Below 550 cm^{-1} deformation modes of the Cu-O coordination octahedra are found, whereas δ(OSO), δ(OBO), and δ(SOB) are present up to around 900 cm^{-1}. Above 900 cm^{-1} stretching modes are dominant: υ(BO) vibrations are found between 910 and 1125 cm^{-1} and vibrations tend to have lower energies than υ(SO), which appear between 930 and 1282 cm^{-1}. IR active stretching modes are mostly asymmetric. For the Raman spectrum of $Cu[B_2(SO_4)_4]$ no intensities were obtained but vibrational mode wavenumbers were indicated by lines in Figure S10. An assignment of all calculated modes is found in Table S13. While bending and stretching modes are found in similar regimes as in the IR spectra, in contrast, Raman active υ(BO) and υ(SO) are mostly symmetric.

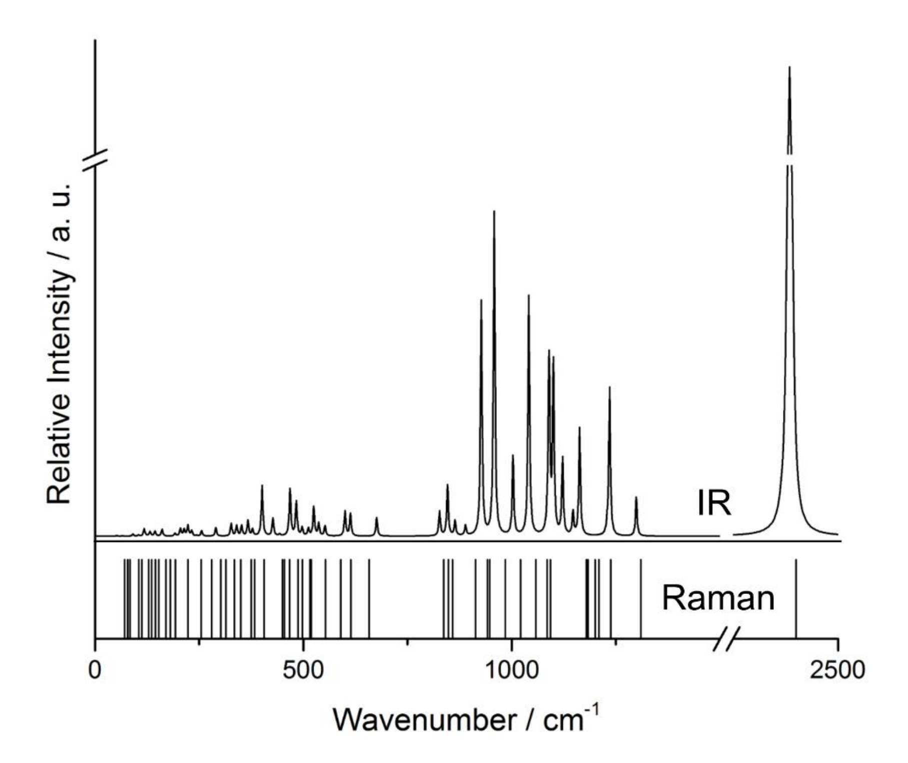

Figure S10. Calculated IR spectra of $Cu[B(SO_4)_2(HSO_4)]$. Intensities are normalized to the most intense peak. Lorentzian line shape profiles were used to generate the spectra using *J-ICE* [2].

For $Cu[B(SO_4)_2(HSO_4)]$, 105 vibrational modes were found, whereof 54 are IR active and 51 modes Raman active. Out of these 105 vibrational modes, the symmetry representation analysis of the Γ-point revealed the following composition: $\Gamma=51A_g+54A_u$. IR and Raman active modes were found between 52 and 2460 cm^{-1} as may be seen from the convoluted IR spectra with Lorentzian shape line profiles and the Raman line spectra depicted in Figure S10. Similar to $Cu[B_2(SO_4)_4]$, bending modes

prevail < 900 cm^{-1} and stretching modes between 900 and 2460 cm^{-1}. Below 400 cm^{-1} deformation vibrations of the Cu-O octahedra are found in the IR and Raman spectra, whereas between 400 and 900 cm^{-1} almost exclusively δ(OSO), δ(OBO), and δ(SOB) modes are visible. Starting from 846 cm^{-1} up to 1310 cm^{-1}, υ(SOH) stretching and δ(SOH) bending modes are found. υ(BO) vibrations are in the regime between 913 and 1147cm^{-1} and υ(SO) modes between 947 and 1238 cm^{-1}. Very distinct are the O-H stretching vibrations, the IR active υ_{sym}(OH) mode at 2454 cm^{-1} and the Raman active υ_{asym}(OH) mode at 2460 cm^{-1}. A complete assignment of all IR and Raman modes is listed in Table S14.

Table S10. Unit cell and structural parameters of $Cu[B_2(SO_4)_4]$ and $Cu[B(SO_4)_2(HSO_4)]$ for the experimental crystal structures as well as for the PBESOL and HSESOL geometry optimized systems.

	$Cu[B_2(SO_4)_4]$			$Cu[B(SO_4)_2(HSO_4)]$	
	Exp.	PBESOL	HSESOL	Exp.	HSESOL
a / Å	5.2470(3)	5.330	5.294	5.3096(3)	5.379
b / Å	7.1371(3)	7.432	7.333	7.0752(4)	7.159
c / Å	7.9222(5)	8.073	8.074	11.2977(6)	11.451
α / °	73.814(3)	72.671	72.989	81.154(1)	81.409
β / °	70.692(2)	70.616	70.060	80.302(2)	81.221
γ / °	86.642(2)	85.162	85.391	80.897(4)	80.408
V / Å^3	268.71(3)	287.940	281.697	409.54(4)	426.195
ρ / g cm^{-3}	2.90	2.703	2.763	2.95	2.827

Table S11. Hirshfeld charge analysis of $Cu[B_2(SO_4)_4]$ and $Cu[B(SO_4)_2(HSO_4)]$ calculated for HSESOL (for details see Quantum Chemical Methodology).

	$Cu[B_2(SO_4)_4]$ HSESOL	$Cu[B(SO_4)_2(HSO_4)]$ HSESOL
Cu	1.00	1.00 – 1.03
S	2.30 – 2.35	2.31 – 2.32

O	-0.70 – -0.95	-0.73 – -0.90
B	1.50	1.50
H	-	0.53

Table S12. Hirshfeld spin density analysis of $Cu[B_2(SO_4)_4]$ and $Cu[B(SO_4)_2(HSO_4)]$ calculated for HSESOL (for details see Quantum Chemical Methodology).

	$Cu[B_2(SO_4)_4]$ HSESOL	$Cu[B(SO_4)_2(HSO_4)]$ HSESOL
Cu	0.77	0.078
S	0 – 0.01	0 – 0.01
O	0 – 0.05	0 – 0.06
B	0	0
H	0	0

Table S13. Calculated IR and Raman frequencies as well as IR intensities of $Cu[B_2(SO_4)_4]$. Results were obtained for the HSESOL optimized structure.

Wavenumber/ cm^{-1}	Irrep.	IR	Intensity	Raman	Mode

75.19	A_g	I	0	A	δ(OSO)
93.42	A_g	I	0	A	δ(CuOS)
94.59	A_u	A	7828.8	I	δ(OSO)
99.83	A_g	I	0	A	δ(OSO)
111.54	A_u	A	24709.9	I	δ(OSO) / δ(CuOS)
116.40	A_g	I	0	A	δ(OSO) / δ(OCuO)
131.84	A_g	I	0	A	δ(OCuO)
137.80	A_u	A	333.7	I	δ(OSO)
152.18	A_u	A	70718.4	I	δ(CuOS)
160.54	A_g	I	0	A	δ(OBO)
186.26	A_u	A	152364.7	I	δ(OCuO)
193.43	A_g	I	0	A	δ(OBO)
203.35	A_u	A	8979.6	I	δ(OBO) / δ(OSO)
218.07	A_u	A	39990.3	I	δ(OCuO)
233.56	A_g	I	0	A	δ(OBO) / δ(OCuO)
247.34	A_g	I	0	A	δ(OSO)
260.33	A_u	A	85.6	I	δ(OSO)
284.85	A_u	A	397.7	I	δ(OSO)
300.78	A_u	A	1375.0	I	δ(OBO) / δ(OSO)
301.77	A_g	I	0	A	δ(OSO) / δ(OCuO)
316.49	A_g	I	0	A	δ(OSO) / δ(CuOS)
326.05	A_u	A	28734.2	I	δ(OBO) / δ(OSO) / δ(OCuO)
342.77	A_g	I	0	A	δ(OSO)
352.91	A_u	A	11327.2	I	υ_{asym}(CuO)

366.42	A_u	A	13745.5	I	δ(CuOS)
369.37	A_g	I	0	A	δ(OSO)
399.73	A_g	I	0	A	δ(OSO)
415.54	A_u	A	438552.5	I	δ(OBO) / δ(OCuO)
442.40	A_g	I	0	A	δ(OSO)
453.90	A_u	A	79339.0	I	δ(OSO)
461.00	A_g	I	0	A	δ(OSO)
466.25	A_u	A	181385.7	I	δ(OBO)
468.22	A_g	I	0	A	δ(OSO)
487.01	A_u	A	241782.2	I	δ(OSO)
499.99	A_g	I	0	A	δ(OSO)
500.63	A_u	A	21468.4	I	δ(OSO)
539.85	A_u	A	21151.7	I	δ(OSO) / δ(OCuO)
546.54	A_g	I	0	A	δ(OSO)
560.72	A_g	I	0	A	δ(SOB) / δ(OBO)
566.85	A_u	A	25642.2	I	δ(SOB) / δ(OBO)
585.95	A_g	I	0	A	δ(SOB)
593.64	A_u	A	23136.2	I	δ(OSO)
646.76	A_g	I	0	A	δ(SOB)
675.58	A_u	A	152127.4	I	δ(SOB) / δ(OSO)
817.95	A_g	I	0	A	δ(OBO)
820.14	A_u	A	6702.3	I	δ(OBO)
838.67	A_u	A	9502.1	I	δ(SOB) / δ(OBO)
909.59	A_g	I	0	A	υ_{sym}(BO)

919.62	A_u	A	488557.0	I	δ(OBO)
921.59	A_g	I	0	A	δ(OBO) / υ_{sym}(SO)
929.51	A_u	A	103030.1	I	υ_{asym}(BO)
936.03	A_g	I	0	A	δ(SOB) / δ(OBO) / υ_{sym}(SO)
988.23	A_g	I	0	A	δ(OBO)
1002.08	A_g	I	0	A	υ_{asym}(BO) / υ_{sym}(SO)
1020.39	A_u	A	2455874.0	I	υ_{asym}(SO)
1038.13	A_g	I	0	A	υ_{sym}(SO)
1039.14	A_u	A	3941.9	I	υ_{asym}(BO)
1093.59	A_u	A	580238.7	I	δ(SOB) / υ_{asym}(BO) / υ_{sym}(SO)
1111.04	A_g	I	0	A	υ_{sym}(BO) / δ(OBO)
1125.40	A_u	A	5846022.3	I	υ_{sym}(BO)
1143.90	A_u	A	1033720.2	I	υ_{asym}(BO) / υ_{asym}(SO)
1163.88	A_g	I	0	A	υ_{sym}(SO)
1252.66	A_u	A	167376.5	I	υ_{asym}(SO)
1261.32	A_g	I	0	A	υ_{sym}(SO)
1281.70	A_u	A	5086032.4	I	υ_{asym}(SO)
1286.16	A_g	I	0	A	υ_{sym}(SO)

Table S14. Calculated IR and Raman frequencies as well as IR intensities of $Cu[B(SO_4)_2(HSO_4)]$ obtained with HSESOL.

Wavenumber/ cm^{-1}	Irrep.	IR	Intensity	Raman	Mode

51.80	A_u	A	6.57	I	δ(OSO)
65.90	A_u	A	5.45	I	δ(CuOS)/ δ(SOB)
70.56	A_g	I	0	A	δ(CuOS)
78.45	A_g	I	0	A	δ(OSO)
83.74	A_g	I	0	A	δ(OCuO)
90.86	A_u	A	26.2	I	δ(CuOS)
103.84	A_u	A	14.31	I	δ(OSO)
105.20	A_g	I	0	A	δ(CuOS)
111.88	A_g	I	0	A	δ(OSO)
118.03	A_u	A	87.54	I	δ(CuOS)
121.63	A_u	A	4.16	I	δ(CuOS)
128.89	A_g	I	0	A	δ(OSO)
132.01	A_u	A	56.04	I	δ(CuOS)
136.26	A_g	I	0	A	δ(OCuO)/ δ(OSO)
144.09	A_u	A	59.27	I	δ(OCuO)
145.36	A_g	I	0	A	δ(OCuO)
152.69	A_g	I	0	A	δ(SOB)
158.53	A_u	A	5.48	I	δ(OSO)/ δ(OBO)
160.52	A_u	A	80.33	I	δ(OCuO)
170.36	A_g	I	0	A	δ(OSO)
181.24	A_g	I	0	A	δ(OSO)
192.22	A_u	A	32.75	I	δ(CuOS)/ δ(OSO)/ δ(OBO)
192.67	A_g	I	0	A	δ(OCuO)
205.41	A_u	A	92.9	I	δ(OSO)

214.13	A_u	A	82.43	I	δ(CuOS)
222.69	A_u	A	133.11	I	δ(OCuO)
223.28	A_g	I	0	A	δ(OSO)
231.87	A_u	A	65.72	I	δ(OSO)/ δ(OCuO)
244.17	A_u	A	8.3	I	δ(OCuO)
255.11	A_g	I	0	A	δ(OSO)
256.15	A_u	A	65.19	I	δ(OSO)/ δ(OCuO)
280.39	A_g	I	0	A	δ(OSO)/ δ(OCuO)
290.46	A_u	A	101.95	I	δ(OCuO)
301.72	A_g	I	0	A	δ(OCuO)
314.25	A_g	I	0	A	υ_{asym}(OCuO)/ δ(CuOS)
327.25	A_u	A	152.72	I	δ(OSO)/ δ(OCuO)
334.00	A_g	I	0	A	δ(OSO)/ δ(OCuO)
339.66	A_u	A	125.91	I	δ(OSO)/ δ(OCuO)
349.70	A_g	I	0	A	δ(OSO)/ δ(OBO)/ δ(OCuO)
352.26	A_u	A	125.93	I	δ(OSO)/ δ(OCuO)
367.09	A_u	A	189.51	I	δ(OSO)
374.69	A_g	I	0	A	δ(OSO)
378.21	A_u	A	79.57	I	δ(CuOS)
383.09	A_g	I	0	A	δ(OSO)
400.79	A_u	A	622.31	I	δ(CuOS)/ δ(OCuO)
406.08	A_g	I	0	A	δ(OSO)
427.35	A_u	A	219.18	I	δ(OSO)
443.21	A_u	A	20.88	I	δ(OSO)/ δ(OBO)

449.95	A_g	I	0	A	δ(OSO)/ δ(OBO)/ δ(CuOS)
454.65	A_g	I	0	A	δ(OSO)
467.10	A_g	I	0	A	δ(OSO)
467.78	A_u	A	576.66	I	δ(OSO)
482.96	A_u	A	421.95	I	δ(OSO)
486.99	A_g	I	0	A	δ(OSO)
497.30	A_u	A	102.96	I	δ(OSO)
498.15	A_g	I	0	A	δ(OSO)
512.30	A_u	A	91.82	I	δ(OSO)
515.89	A_g	I	0	A	δ(OSO)/ δ(OBO)
518.54	A_g	I	0	A	δ(OSO)/ δ(SOB)
524.78	A_u	A	356.13	I	δ(SOB)
537.39	A_u	A	152.85	I	δ(OSO)/ δ(OBO)
551.64	A_u	A	120.17	I	δ(SOB)
553.34	A_g	I	0	A	δ(OSO)
589.59	A_g	I	0	A	δ(OSO)/ δ(SOB)
599.52	A_u	A	306.41	I	δ(OSO)/ δ(CuOS)
612.97	A_u	A	274.28	I	δ(OSO)/ δ(SOB)
613.94	A_g	I	0	A	δ(SOB)
657.57	A_g	I	0	A	δ(OSO)/ δ(SOB)
676.40	A_u	A	228.68	I	δ(OSO)
827.20	A_u	A	302.94	I	δ(OBO)
836.95	A_g	I	0	A	δ(OBO)
845.71	A_u	A	622.22	I	υ(SOH)

848.13	A_g	I	0	A	υ(SOH)
858.21	A_g	I	0	A	υ(SOH)
864.18	A_u	A	182.29	I	δ(SOH)/ δ(OBO)
889.01	A_u	A	122.83	I	δ(SOH)/ δ(OBO)
912.61	Ag	I	0	A	$υ_{sym}$(BO)
927.25	A_u	A	2887.92	I	$υ_{asym}$(SO)
941.76	A_g	I	0	A	$δ_{sym}$(SOH)
947.37	A_g	I	0	A	$υ_{sym}$(SO)
957.79	A_u	A	3987.66	I	$υ_{asym}$(BO)
984.59	A_g	I	0	A	$υ_{sym}$(BO)/ $υ_{sym}$(SO)
1002.89	A_u	A	972.3	I	$υ_{asym}$(BO)
1020.70	A_g	I	0	A	$υ_{sym}$(BO)
1041.20	A_u	A	2955.52	I	$υ_{asym}$(BO)
1057.55	A_g	I	0	A	$υ_{sym}$(BO)
1085.47	A_g	I	0	A	$υ_{sym}$(BO)
1087.60	A_u	A	607.81	I	$υ_{asym}$(BO)
1089.58	A_u	A	1843.66	I	υ(SO)/ υ(BO)/ $δ_{asym}$(SOH)
1093.27	A_g	I	0	A	δ(OBO)
1099.57	A_u	A	2086.74	I	$υ_{asym}$(SO)
1122.09	A_u	A	938.69	I	$υ_{asym}$(SO)
1147.41	A_u	A	280.55	I	υ(SO)/ υ(BO)
1162.77	A_u	A	1325.56	I	$υ_{sym}$(SO)
1179.20	A_g	I	0	A	$υ_{sym}$(SO)
1182.61	A_g	I	0	A	$υ_{asym}$(SO)

1199.68	A_g	I	0	A	υ_{asym}(SO)
1209.42	A_g	I	0	A	δ_{asym}(SOH)
1229.61	A_u	A	96.14	I	δ_{sym}(SOH)
1234.93	A_u	A	1818.59	I	υ_{asym}(SO)
1237.96	A_g	I	0	A	υ_{sym}(SO)
1298.56	A_u	A	477.12	I	δ_{asym}(SOH)
1310.07	A_g	I	0	A	δ_{sym}(SOH)
2453.67	A_u	A	9739.29	I	υ_{asym}(OH)
2459.51	A_g	I	0	A	υ_{sym}(OH)

Scheme S1. pK_a values were calculated according to the depicted thermodynamic cycle. Instead of direct calculation of the property of interest $\Delta G^{Rxn}{}_{(solv)}$ (depicted by the red arrow), it is calculated *via* the gas phase reaction free energy $\Delta G^{Rxn}{}_{(g)}$ and the solvation free energies of the protonated acid $HA_{(g)}$, $\Delta G_{(solv)}$(HA), and the dissociated products H^+ and A^-, $\Delta G_{(solv)}(H^+)$ and $\Delta G_{(solv)}(A^-)$.

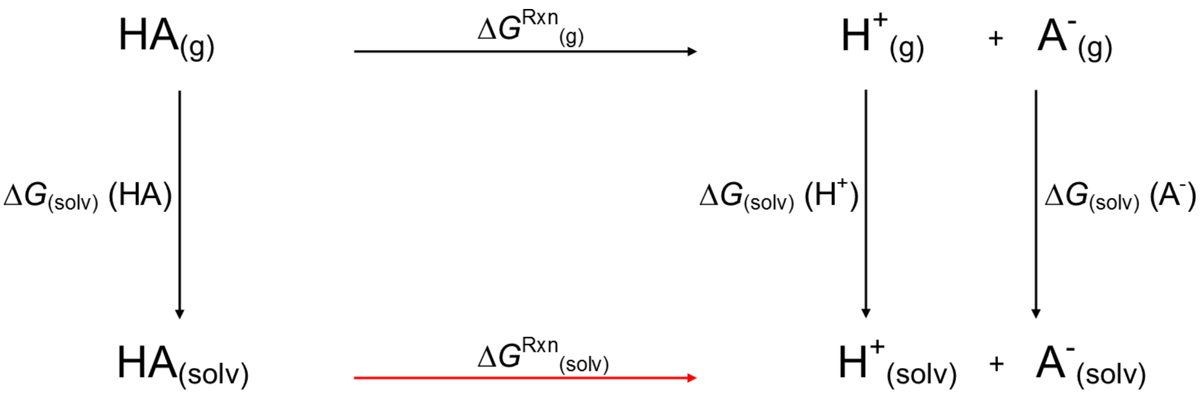

pK_a values of the complex anion $[B_2(SO_4)_4(SO_4H)_2]^{4-}$ in twisted and chair conformation were obtained according to the thermodynamic cycle shown in Scheme S1 from the free energy of solvation for the deprotonation ΔG^{Rxn} (solv) as highlighted by the red arrow. Rather than direct calculation of this property, it is obtained *via* the free energies of solvation for each species, ΔG (solv) (HA), ΔG (solv) (H^+), ΔG (solv) (A^-), and the gas phase reaction free energy ΔG^{Rxn} (g) (shown with black arrows).

Gas phase as well as solvent free energies of H^+, were taken from the literature. The gas phase free energy of H^+ is set to G (g) (H^+) = –26.32 kJ mol^{-1}, using the Sackur-Tetrode equation and translational energy at 298 K [4], whereas the solvent free energy is ΔG (solv) (H^+) = -1087.00 kJ mol^{-1} (Scheme S1) [5].

Table S15. Calculated pK_a values for the Chair and Twist form of the complex oxoanion $[B_2(SO_4)_4(SO_4H)_2]^{4-}$ as well as H_2SO_4 and $Si(OH)_4$.

	**Chair**		**Twist**	
	BP86/def2-TZVP	PBE0/cc-pVTZ	BP86/def2-TZVP	PBE0/cc-pVTZ
pK_a1	6.6	9.2	6.2	9.8
pK_a2	9.4	*.	8.1	11.9
	**H_2SO_4**		**$Si(OH)_4$**	
	BP86/def2-TZVP	PBE0/cc-pVTZ	BP86/def2-TZVP	PBE0/cc-pVTZ
pK_a	-5.3	-1.9	17.5	23.7

*PBE/cc-pVTZ calculation of the chair conformation did not converge.

As the calculation of pK_a values with DFT is known to be sensitive to the methodology [3], for all pK_a calculations two different density functionals were tested: the BP86 density functional [5], together with the resolution of identity technique [6], and the PBE0 [7] density functional were used in combination with the def2-TZVP basis set of triple-zeta quality in the case of BP86 [8] and the correlation-consistent cc-pVTZ basis set in the case of PBE0 [9]. For sake of comparison, the pK_a values of sulfuric acid and silicic acid were also calculated. Although absolute pK_a values differ between the two calculations, the same relative trend is observed: the borosulfate anion has a pK_a that is in between that of a strong acid such as H_2SO_4 and that of $Si(OH)_4$.

Magnetochemical Investigation

Figure S11. Magnetic behavior of $Cu[B(SO_4)_2]$: magnetic susceptibility measured in ZFC mode at 10 kOe (*top*); magnetization isotherms recorded at 3, 10 and 50 K (*bottom*).

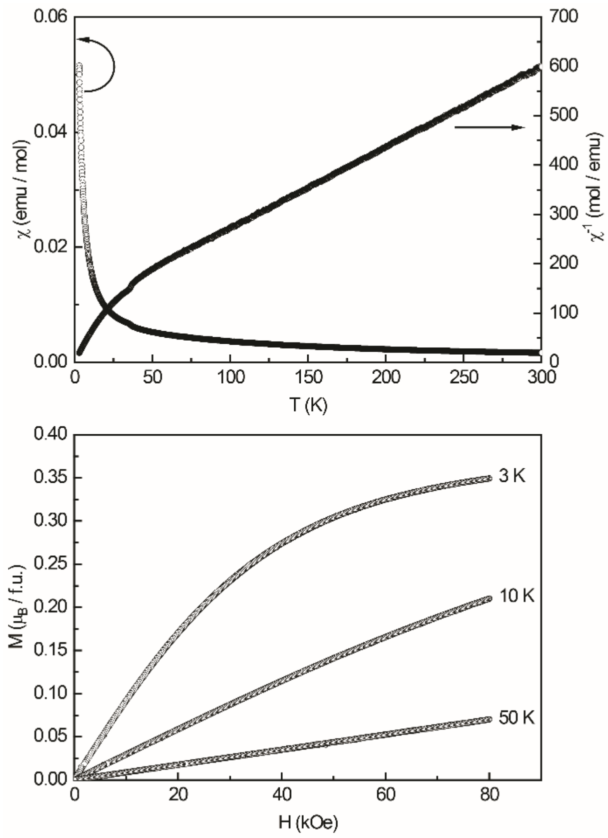

Figure S11 depicts the magnetic measurements of the mixture of Cu[$B_2(SO_4)_4$] and Cu[$B(SO_4)_2(HSO_4)$]. The top panel shows the zero-field cooled curve measured at 10 kOe. The top panel shows no ordering down to low temperatures along with traces of impurities, causing the bump at 35 K. Magnetization isotherms shown in the bottom panel indicate that paramagnetic compounds are present.

References

[1] a) R. Demichelis, B. Civalleri, M. Ferrabone, R. Dovesi, *Int. J. Quantum Chem.* **2010**, *110*, 406-415; b) B. Montanari, B. Civalleri, C. M. Zicovich-Wilson, R. Dovesi, *Int. J. Quantum Chem.* **2006**, *106*, 1703-1714; c) C. M. Zicovich-Wilson, F. Pascale, C. Roetti, V. R. Saunders, R. Orlando, R. Dovesi, *J. Comput. Chem.* **2004**, *25*, 1873-1881.

[2] P. Canepa, R. M. Hanson, P. Ugliengo, M. Alfredsson *J. Appl. Crystallogr.* **2011**, *44*, 225-229.

[3] See e.g., J. Ho, M. L. Coote, *Wiley Interdiscip. Rev. Comput. Mol. Sci.* **2011**, *1*, 649−660, and references therein.

[4] P. Hünenberger, M. Reif, *Single-Ion Solvation. Experimental and Theoretical Approaches to Elusive Thermodynamic Quantities*. RSC Publishing, London, **2011**.

[5] a) A. D. Becke, *Phys. Rev. A.* **1988**, *38*, 3098-3100; b) J. P. Perdew, *Phys. Rev. B* **1986**, *33*, 8822-8824.

[6] K. Eichkorn, O. Treutler, H. Öhm, M. Häser, R. Ahlrichs, *Chem. Phys. Lett.* **1995**, *240*, 283-290.

[7] J. P. Perdew, K. Burke, M. Ernzerhof, *Phys. Rev. Lett.* **1996**, *77*, 3865–3868.

[8] F. Weigend, R. Ahlrichs, *Phys. Chem. Chem. Phys*. **2005**, *7*, 3297-3305.

[9] T. H. Dunning, *J. Chem. Phys.* **1989**, 1007-1023.
